# Apalutamide-Induced Pneumonitis Presenting as Severe Hypoxemic Respiratory Failure in Metastatic Prostate Cancer: A Case Report

**DOI:** 10.7759/cureus.111552

**Published:** 2026-06-26

**Authors:** Tashfeen Mahmood, Mohammad M Mahmood, Elizabeth Martin

**Affiliations:** 1 Pulmonary, Critical Care, and Sleep Medicine, CHRISTUS Ochsner St. Patrick Hospital, Lake Charles, USA; 2 Science, Bishop Noland Episcopal Day School, Lake Charles, USA

**Keywords:** androgen receptor inhibitor, apalutamide, drug-induced pneumonitis, interstitial lung disease, prostate cancer, pulmonary toxicity

## Abstract

Apalutamide is a widely used second-generation androgen receptor inhibitor for the treatment of metastatic castration-sensitive and nonmetastatic castration-resistant prostate cancer. Although generally well tolerated, rare cases of interstitial lung disease and drug-induced pneumonitis have been reported. Early recognition is critical because of the potential for significant morbidity and mortality. We present a case of suspected apalutamide-induced pneumonitis in a 69-year-old man with metastatic prostate cancer who developed progressive shortness of breath, severe hypoxia, and bilateral ground-glass opacities on imaging. Extensive evaluation excluded infectious, cardiac, and autoimmune etiologies. The patient demonstrated marked clinical and radiographic improvement following the discontinuation of apalutamide and corticosteroid therapy. This case highlights the importance of maintaining a high index of suspicion for drug-induced pulmonary toxicity in patients receiving apalutamide. Prompt diagnosis and early initiation of corticosteroids may significantly improve outcomes.

## Introduction

Apalutamide is a second-generation androgen receptor inhibitor approved for the treatment of metastatic castration-sensitive and nonmetastatic castration-resistant prostate cancer [1,2]. While clinical trials have demonstrated a generally favorable tolerability profile, rare cases of interstitial lung disease (ILD) and drug-induced pneumonitis have been reported in post-marketing surveillance data [3,4]. Apalutamide-associated pneumonitis is rare, occurring in fewer than 1% of treated patients, but it may result in severe respiratory failure requiring hospitalization and prompt drug discontinuation [2]. Drug-induced pneumonitis is a potentially life-threatening condition that often presents with nonspecific symptoms such as dyspnea and hypoxia, making early diagnosis challenging. Recognition is particularly important in oncology patients, in whom overlapping etiologies such as infection, malignancy progression, and cardiac dysfunction must be excluded.

We present a case of suspected apalutamide-induced pneumonitis manifesting as severe hypoxemic respiratory failure. The diagnosis was made after alternative etiologies were excluded, and significant clinical improvement was observed following discontinuation of apalutamide and initiation of corticosteroid therapy.

## Case presentation

A 69-year-old African American man with a 20-pack-year smoking history presented with progressively worsening shortness of breath over several days (Table 1). The patient had no known history of ILD, connective tissue disease-associated lung disease, or other chronic parenchymal lung disorders. Although prior chest imaging was not available for review, he had no documented history of chronic respiratory symptoms, oxygen requirement, or previously identified radiographic abnormalities suggestive of preexisting ILD. He had previously received degarelix without pulmonary adverse effects, arguing against gonadotropin-releasing hormone antagonist-related toxicity. The patient had been receiving apalutamide 240 mg orally once daily for metastatic prostate cancer, initiated approximately 10 weeks before presentation.

**Table 1:** Summary of key clinical and imaging findings at presentation

Clinical course	Key findings	Figure/notes
Presentation	Progressively worsening dyspnea over several days. Hypoxic on room air, requiring nasal cannula/Oxymizer and later high-flow oxygen with AIRVO.	Reported in text
CT chest with contrast on presentation	Diffuse bilateral ground-glass opacities with intra- and interlobular septal thickening and small bilateral pleural effusions.	Figure 1
Echocardiogram	Normal biventricular function with a left ventricular ejection fraction of 60-65%. No significant valvular abnormalities.	Reported in text
CT chest without contrast after initiation of high-dose systemic corticosteroids	Significant improvement in diffuse ground-glass opacities and interlobular septal thickening.	Figure 2

**Figure 1:**
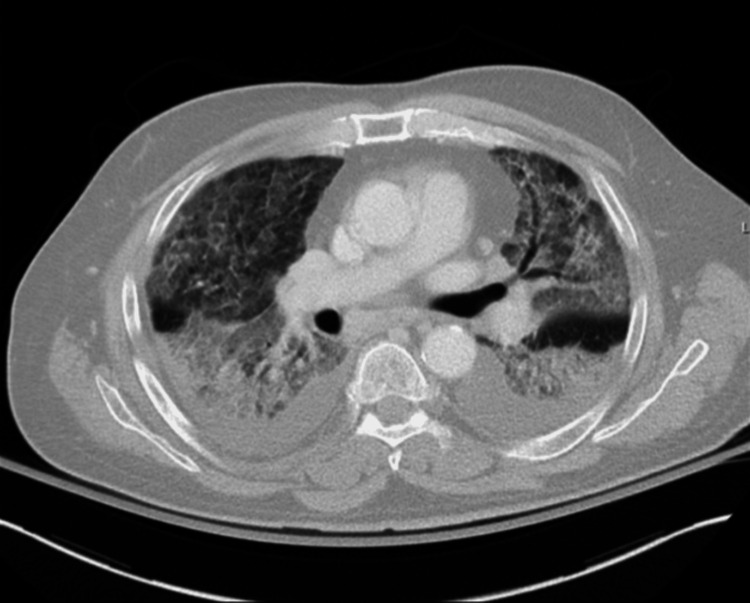
CT chest on presentation demonstrating diffuse bilateral ground-glass opacities and interlobular septal thickening

**Figure 2:**
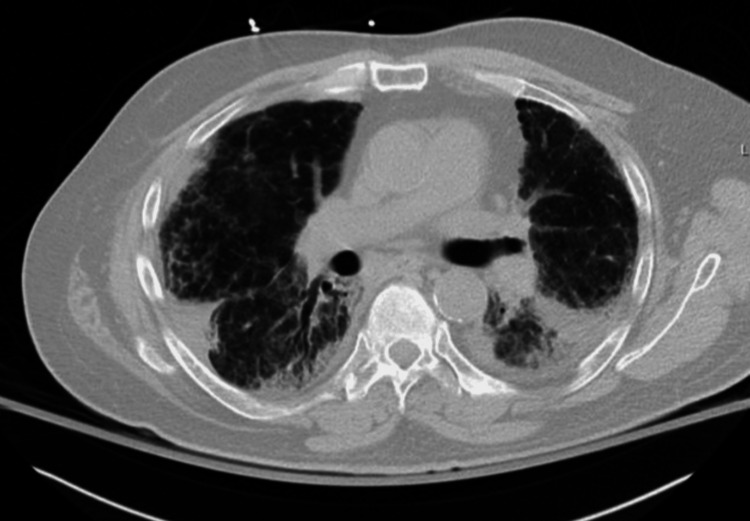
Follow-up CT chest showing significant improvement in ground-glass opacities and interlobular septal thickening

Physical examination demonstrated no jugular venous distention, peripheral edema, Raynaud phenomenon, Gottron papules, heliotrope rash, mechanic’s hands, digital ulcerations, or other findings suggestive of connective tissue disease or pulmonary hypertension.

On admission, the patient required 5 L/min of oxygen, which progressively increased to 15 L/min via Oxymizer, prompting pulmonary consultation. Oxygen saturation ranged from 92% to 94%, with tachycardia at 125 beats per minute. Arterial blood gas analysis revealed severe hypoxemia, with a PaO₂ of 60 mmHg on 15 L/min of oxygen delivered via Oxymizer (Table 2). The calculated PaO₂/FiO₂ ratio was estimated to be approximately <100, consistent with severe hypoxemic respiratory failure. The patient’s condition subsequently deteriorated, and he was placed on an AIRVO high-flow nasal cannula system, requiring an FiO₂ of 95% at a flow rate of 60 L/min.

**Table 2:** Pertinent initial laboratory findings ANCA, antineutrophil cytoplasmic antibody; BNP, B-type natriuretic peptide; MRSA, methicillin-resistant *Staphylococcus aureus*

Parameter	Value	Reference range
WBC	13.0 × 10³/µL	4.6-10.2 × 10³/µL
Arterial blood gas	pH 7.46; PaCO₂ 37.5 mmHg; PaO₂ 60 mmHg on 15 L/min via Oxymizer	pH 7.35-7.45; PaCO₂ 35-45 mmHg; PaO₂ 80-100 mmHg
BNP	51.3 pg/mL	0.0-100.0 pg/mL
Erythrocyte sedimentation rate	115 mm/h	0-15 mm/h
ANCA screen	Negative	<1:20 normal; >1:20 abnormal
D-dimer	2,316 ng/mL FEU	0-500 ng/mL FEU
Procalcitonin	0.22 ng/mL	0.00-0.50 ng/mL
Lactate	1.6 mmol/L	0.4-2.0 mmol/L
Antinuclear antibody screen	None detected	None detected
Rheumatoid factor qualitative screen	Negative	Negative
Legionella antigen, urine	Negative	Negative
*Streptococcus pneumoniae* antigen, urine	Negative	Negative
MRSA cultures, nares (left and right), and respiratory	No growth	+
Respiratory pathogen panel, PCR - nasopharyngeal		
Adenovirus	Not detected	Not detected
Coronavirus 229E	Not detected	Not detected
Coronavirus HKU1	Not detected	Not detected
Coronavirus NL63	Not detected	Not detected
Coronavirus OC43	Not detected	Not detected
SARS-CoV-2 (COVID-19)	Not detected	Not detected
Human metapneumovirus	Not detected	Not detected
Human rhinovirus/enterovirus	Not detected	Not detected
Influenza A	Not detected	Not detected
Influenza B	Not detected	Not detected
Parainfluenza 1	Not detected	Not detected
Parainfluenza 2	Not detected	Not detected
Parainfluenza 3	Not detected	Not detected
Parainfluenza 4	Not detected	Not detected
Respiratory syncytial virus	Not detected	Not detected
Bordetella parapertussis	Not detected	Not detected
Bordetella pertussis	Not detected	Not detected
Chlamydia pneumoniae	Not detected	Not detected
Mycoplasma pneumoniae	Not detected	Not detected

CT angiography of the chest demonstrated no pulmonary embolism but showed diffuse bilateral ground-glass opacities with interlobular septal thickening and small pleural effusions, suggestive of atypical infection, pulmonary edema, or pneumonitis (Figure 1).

An ECG obtained during the initial evaluation showed sinus tachycardia. No ECG findings explained the patient’s severe hypoxemic respiratory failure (Figure 3).

**Figure 3:**
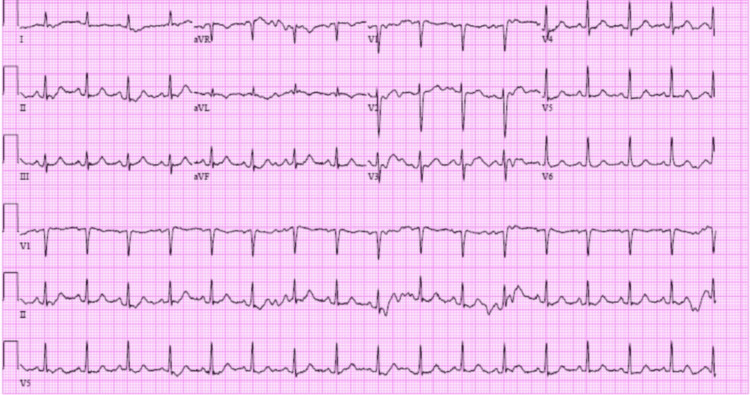
Admission ECG demonstrating sinus tachycardia without acute ischemic changes

The infectious workup was unrevealing, with negative results for the respiratory pathogen panel, urinary antigens for *Legionella *and *Streptococcus pneumoniae*, and respiratory cultures. Procalcitonin was within normal limits at 0.22 ng/mL. Autoimmune evaluation, including ANA, RF, and ANCA, was negative, effectively excluding connective tissue disease-associated ILD. BNP was normal at 51.3 pg/mL, and echocardiography confirmed preserved biventricular function with a left ventricular ejection fraction of 60-65%, arguing against cardiogenic pulmonary edema. D-dimer was elevated; however, CTA of the chest excluded pulmonary embolism. The erythrocyte sedimentation rate was elevated but nonspecific and interpreted in the context of underlying malignancy and systemic inflammation (Tables 1, 2). Bronchoalveolar lavage and transbronchial biopsy were not pursued because of severe hypoxemic respiratory failure, concern for procedural deterioration, and the high clinical suspicion for drug-induced pneumonitis.

Given the exclusion of infectious, cardiac, and autoimmune etiologies, apalutamide-induced pneumonitis with severe hypoxemic respiratory failure was strongly suspected [3,5]. The patient was initiated on high-dose corticosteroids with methylprednisolone 1000 mg IV daily for three days, followed by 60 mg IV every eight hours, resulting in progressive clinical improvement. Methylprednisolone was subsequently transitioned to prednisone 60 mg orally daily for one week, and the patient was transferred to a long-term acute care facility on prednisone 40 mg orally daily, where gradual oxygen tapering was performed over three weeks. Steroids were tapered by 10 mg per week from this dose. Follow-up CT imaging demonstrated significant resolution of pulmonary infiltrates (Figure 2).

Application of the Naranjo Adverse Drug Reaction Probability Scale yielded a score of 7, consistent with a probable adverse drug reaction [6]. The patient’s oncology team at a tertiary care center was contacted and updated regarding his diagnosis and clinical course. Rechallenge with apalutamide was not recommended. The patient was ultimately discharged home on room air with complete clinical recovery. Further oncologic follow-up was unavailable because the patient subsequently relocated out of state.

## Discussion

Apalutamide-associated pneumonitis is a rare but potentially life-threatening complication. The precise mechanism of apalutamide-associated pulmonary toxicity is not well established. The leading hypothesis is a delayed hypersensitivity or immune-mediated reaction rather than direct cytotoxicity [4].

Kobe et al. described two cases of severe apalutamide-induced ILD requiring corticosteroid therapy, emphasizing the potential severity of this adverse effect [3]. In a separate report, Kirishima et al. documented a case in a patient with castration-resistant prostate cancer, underscoring the diagnostic challenge posed by overlapping etiologies and the need for early intervention [4].

Drug-induced pneumonitis typically presents with nonspecific symptoms such as progressive dyspnea and hypoxia, accompanied by bilateral ground-glass opacities on imaging. Diagnosis remains largely one of exclusion, requiring careful evaluation to rule out infection, autoimmune disease, malignancy progression, and cardiogenic etiologies.

In this patient, symptoms developed approximately 10 weeks after initiation of therapy. Although histopathologic confirmation was not obtained, the diagnosis was supported by the temporal relationship between apalutamide exposure and symptom onset, characteristic radiographic findings, and marked clinical and radiographic improvement following drug discontinuation and initiation of corticosteroid therapy. Alternative etiologies were systematically excluded, including infectious pneumonia, cardiogenic pulmonary edema, connective tissue disease-associated ILD, pulmonary embolism, preexisting ILD, and other medication-related pulmonary toxicity, based on clinical, laboratory, and imaging findings. This diagnostic approach is consistent with previously published cases of apalutamide-associated ILD [3,4].

This case reinforces the importance of maintaining a high index of suspicion for pulmonary toxicity in patients receiving apalutamide. To our knowledge, only a limited number of well-described cases have been reported in the English-language literature. Early recognition and prompt corticosteroid therapy are critical to improving outcomes and preventing progression to severe respiratory failure. Continued reporting of such cases is essential to better characterize the incidence, clinical course, and optimal management of apalutamide-associated pulmonary toxicity.

## Conclusions

Apalutamide-induced pneumonitis, although rare, is a clinically significant complication that requires early recognition and prompt management. Clinicians should remain vigilant for respiratory symptoms in patients receiving second-generation androgen receptor inhibitors, particularly apalutamide, as early diagnosis and initiation of corticosteroid therapy can lead to favorable outcomes.
